# Adoptive T Cell Therapy Targeting MAGE-A4

**DOI:** 10.3390/cancers17030413

**Published:** 2025-01-26

**Authors:** Kapil Chandora, Akshay Chandora, Anwaar Saeed, Ludimila Cavalcante

**Affiliations:** 1 Morehouse School of Medicine, 720 Westview Dr, Atlanta, GA 30310, USA; kchandora@msm.edu (K.C.);; 2 UPMC Hillman Cancer Center, University of Pittsburgh, Pittsburgh, PA 15232, USA; saeeda3@upmc.edu; 3 Division of Hematology and Oncology, University of Virginia Comprehensive Cancer Center, Charlottesville, VA 22903, USA

**Keywords:** MAGE-A4, cancer testis antigen, immunotherapy, antibody, adoptive T cell therapy, sarcoma, ovarian cancer, esophageal cancer, solid tumors

## Abstract

MAGE A4 (Melanoma Antigen Gene A4) is a cancer testis antigen (CTA) that is expressed normally in germline cells (testis/embryonic tissues) but absent in somatic cells. The MAGE A4 CTA is expressed in a variety of tumor types, like synovial sarcoma, ovarian cancer and non-small cell lung cancer. Having its expression profile limited to germline cells has made MAGE A4 a sought-after immunotherapeutic target in certain malignancies. In this review, we focus on MAGE-A4’s function and expression, current clinical trials involving targeted immunotherapy approaches, and challenges and opportunities facing MAGE-A4’s targeted therapeutics.

## Simple Summary

MAGE A4 is a protein found in several types of cancer, but not usually in healthy tissues. This makes it a good target for therapies that aim to destroy cancer cells while leaving healthy cells unharmed. One promising new treatment is called MAGE A4-targeted autologous T cell genetic therapy. “Autologous” means the therapy uses the patient’s own cells. Doctors take some of the patient’s T cells (a type of immune cell) and genetically modify them to recognize and attack cells that have the MAGE A4 protein. These modified T cells are then grown in large numbers in the lab and given back to the patient. The goal is for these engineered T cells to circulate throughout the body, find the cancer cells with MAGE A4, and eliminate them. Early clinical trials using this approach have shown promising results, with some patients experiencing tumor shrinkage. While there are side effects, this type of therapy offers hope for patients with MAGE A4-positive cancers that haven’t responded to other treatments. In this review, we aim to explain the drawbacks and advantages to this genetic therapy, and explore the current advances in this field.

## 1. Introduction

The therapeutic utilization of T cells against tumor cells is limited by several well-known challenges, such as the need for expansion of tumor antigen-specific T cells to sufficient numbers and the need to target specific sites while overcoming a tumor’s immune tolerance [1]. Some of the earliest applications of T cells in cellular therapy involved extracting tumor-specific T cells from patients with melanoma in a process of adoptive cell transfer [2]. These so-called “tumor infiltrating lymphocytes (TILs)” were stimulated by immunomodulators and expanded in vitro before being reinserted back into the patient [2]. The pursuit of overcoming a tumor’s immune tolerance has led to the genetic engineering of T cell receptors (TCR-Ts) and chimeric antigen receptor T cells (CARTs). Many unique methods have been utilized to create TCRs that are specific to certain cancer types. One of these methods involves using allogeneic T cells from individuals who are intolerant of a patient’s tumor-associated antigens (TAAs) and then incubating them with tumor cells from the patient, allowing for those T cells to be primed against the tumor cells of the patient [3]. Another method involves generating large DNA libraries of TCR genes via bacteriophages that bind to targets of interest in tumors [4]. Some of the earliest successes of TCR therapy were seen in 2006, where MART-1 (Protein melan-A), a TAA in melanoma, was targeted by TCR-Ts and demonstrated tumor regression [5]. Compared to TCR therapy, which can target intracellular tumor antigens, CART therapy involves the targeting of extracellular antigens via antibody fragments. Because CARTs target extracellular antigens, their efficacy is limited when treating solid malignancies, which are normally not present in somatic tissues [3,6]. What separates CART therapy from the use of engineered TCRs is that CART therapy is not MHC-restricted, unlike TCR therapy, which requires HLA matching between TCRs and cancer cells. Despite this issue, CART therapy has been shown to be extremely effective in hematological malignancies, as evidenced by two therapies (tisagenlecleucel and axicabtagene ciloleucel) targeting CD19 which have received FDA approval and have been used to treat thousands of patients with B cell malignancies, with rates of remission exceeding 80% in some populations [7]. Another important consideration is that hematologic malignancies typically exhibit more uniform distributions of antigens, whereas solid tumors often show heterogenous antigen distributions, with varying numbers of antigens at different tumor sites. As a result, the effectiveness of CART cell therapy can be limited by the quality of antigen binding in solid tumors [8]. However, a study by Larson et al. (2022) suggests that this can be overcome by IFNγ. The IFNγ-dependent bystander effect is predominantly observed in solid tumors and not in hematological malignancies [9]. Ultimately, the IFNγ-dependent bystander killing effect for solid tumors relies upon the tumor microenvironments in solid tumors, as their environments can have confined spaces of stromal, cancer and extracellular matrix components that can enable greater concentrations of cytokines like IFNγ, allowing for increased ICAM-1 (intercellular adhesion molecule) expression on neighboring tumor cells and increased CART cell engagement [9,10]. In summary, further understanding of these intricacies in the tumor microenvironment is something that can improve the deficit of CART therapy.

Compared to CART therapy, autologous engineered TCRs (TCR-Ts) allow for targeting of intracellular peptides like cancer testis antigens (CTAs), as they are presented by antigen-presenting cells (APCs) via the major histocompatibility complex (MHC) to TCRs [11]. CTAs have been found to be expressed in multiple tumor types early on, in multiple brain tumors, breast cancers and melanomas [12]. A unique benefit of CTAs is that they are expressed in germline cells (testis or placenta) that cannot express MHC class 1 molecules and have restricted expression in somatic tissue [13]. The expression of CTAs is controlled by epigenetic conditions via DNA methylation and histone post-translational changes; for example, hypomethylation of the promoter sites for CTAs in melanomas is the reason for their heterogeneous intracellular expression [14]. One of the oldest discovered CTAs reported is the MAGE (Melanoma Antigen Gene) family, which includes 17 different genes on the X chromosome that have been found to be expressed in many tumor types, such as breast, prostate, brain, colon, melanoma and lung cancers [15,16,17,18]. The highest number of CTAs are found on the X chromosome in the Xq24-q28 region, which include NYESO-1, MAGE-A and MAGE C [19]. MAGE proteins have also been used as predictive markers, as seen in one study, where the depletion of MAGE A3 in MAGE A3-positive pancreatic cells led to reduced cell proliferation and increased apoptosis as a response to cytotoxic drugs [16].

In another study, MAGE A4 and another CTA, NY-ESO-1, were identified as poor prognostic markers when they were found to be expressed in patients with non-small cell lung cancer (NSCLC), and the presence of both of these CTAs demonstrated not only poor outcomes but also the appearance of myeloid-derived suppressor cells (MDSCs), which are key in evading immune detection [20]. Another instance of CTAs used as predictive markers was in mucosal melanoma patients, where the levels of expression of NY-ESO-1, MAGE-A4 and MAGE A3 correlated with the effectiveness of the immunotherapy combination of ipilimumab and nivolumab [21]. Given the presence of CTAs in multiple cancer types, their role in tumor-driving cellular mechanisms and use as markers of therapeutic benefit make them advantageous targets for autologous TCR therapy. Success in targeting CTAs has been seen in studies with NY-ESO-1-specific TCR cells; a clinical trial by Robbins et al. 2011 demonstrated an objective response rate of 55% in melanoma patients [22]. Another trial (NCT03686124), which targeted HLA-A*02-restricted CTA PRAMEs (Preferentially Expressed Antigens in Melanoma), reported that all 12 participating patients achieved either partial response or stable disease (PR or SD) [23].

## 2. MAGE A4 Functionality and the Beginnings of T Cell Therapy Development

The MAGE protein family is subdivided into MAGE A, B and C, and out of these subtypes the MAGE A family has the greatest potential for immunotherapeutic targets as it is present in various cancer types [24]. In one instance, Cabezon et al., 2013, showed MAGE A4 expression at significant levels in triple-negative breast cancer (TNBC) and in Her 2-positive/ER-negative lesions as well [25]. One other example of such a case can be seen in the MAGE A3 presentation in NSCLC, which is associated with increased morbidity and mortality [16]. Another CTA, MAGE A10 is present in a variety of cancers like melanoma, colorectal cancer, NSCLC, breast cancer and urothelial tumors [26,27,28,29]. Besides being excellent immunotherapeutic targets, the MAGE A family is known to influence some tumor oncogenic pathways. The MAGE A CTA family has been shown to affect oncogenesis at the molecular level by its suppression of p53 transcription, leading to prevention of cell cycle arrest [30]. Particularly, MAGE-A4 plays a role in Tran-lesion synthesis (TLS), which permits ongoing DNA synthesis in cells with damaged genomes by recruiting a Y-family DNA polymerase that can assist in repair. In tumors that over-express MAGE A4, the available MAGE-A4 is depleted, and this leads to TLS dysfunction, contributing to future genome instability [31]. Another pathway MAGE A4 affects is the oncogenic activity of gankyrin, which is overexpressed in hepatocellular carcinomas and plays an important role in tumorigenesis. Though the MAGE family CTAs are biochemically similar, MAGE A4 has a C-terminal binding site that makes it unique, allowing it to bind to gankyrin. In mice that were athymic and in which hepatocytes overproducing gankyrin were induced, MAGE A4 showed partial suppression of gankyrin functionality [32,33]. Besides MAGE-A4’s unique biochemical structure and involvement in various oncologic immunotherapeutic pathways, it also has a strong presence in various malignancies. The expression of MAGE-A4 is notable in head and neck squamous cell carcinoma (HNSCC), synovial sarcoma (SS), Reed–Sternberg cells, serous ovarian neoplasms, myxoid/round cell liposarcoma (MRCLS), NSCLC, urothelial cancer, melanoma and gastroesophageal cancer [34,35,36,37,38,39]. Particularly in SS, MAGE A4 is highly expressed along with NY-ESO1, and the presence of both is correlated with advanced clinical stage and necrosis [36,40]. Given MAGE-A4’s versatility as an immunotherapeutic target, there was great interest in the development of various T cell therapy options; however, finding a viable peptide sequence of this TAA was the first challenge to overcome in this process.

The journey of the development of a viable T cell therapy to target MAGE A4 began with initially targeting MAGE A-1, as this was the first successfully cloned human tumor antigen [24]. The earlier process of creating these cytotoxic T lymphocytes (CTLs) targeting MAGE A1 was called T cell epitope cloning and involves two steps. In the first step, CTLs in the peripheral blood of melanoma patients were isolated, then expanded and stimulated in vitro with autologous tumor cells. In the second step, cDNA (complementary DNA) libraries made up of 700,000 cosmids, derived from epitopes of autologous tumor cells, were exposed to these CTLs. The CTLs were then isolated based on their ability to stimulate tumor necrosis factor (TNF) in various pools of cosmids, especially in pools coding for the MAGE A1 antigen [24]. Later on, a process called serological analysis of cDNA expression libraries (SEREX) was developed, and instead of using autologous T cells to be screened against cDNA libraries, antibodies from the patient’s serum were utilized in their place [41]. Eventually, the SEREX process itself was improved upon by utilizing bioinformatic analysis and enhanced detection of CTAs with oligonucleotide microarrays [42,43,44]. To summarize, the CTLs from patients were extracted and then stimulated in an in vitro environment, where they were exposed to either oligonucleotide microarrays or specific generated antibodies generated from CDNA libraries via SEREX in order to find viable TAA targets that could stimulate autologous T cells in vivo. Ultimately, the SEREX method and the microarray technology assisted greatly in the identification of two antigenic peptides of MAGE A4: GVYDGREHTV, displayed by HLA-A2, and SESLKMIF, displayed by HLA-B37 [45]. Both of these peptides are coded by the MAGE 4a and 4b alleles of the MAGE A4 gene. The gene itself was transcribed into an adenoviral vector to transfect dendritic cells (DCs) and identify/stimulate autologous T cells in vitro [45,46,47]. Once the peptide target was elicited for MAGE A4, the journey to design the TCR receptor could be accomplished [48,49].

Through the course of TCR-T development targeting specific CTAs like MAGE-A4, multiple strategies were developed to improve the durability of TCR-T anti-tumor response and effectiveness. Methods like pairSEQ (paired sequencing) have also been developed for identifying peptide targets of TCRs from isolated TILs from patients and have enabled more precision when it comes to TCR binding [50]. TCR binding to CTA targets has been further improved with specific amino acid substitutions on the antigen binding sites of TCRs, which have improved tumor cell recognition [51]. The early studies of adaptive TCR therapy were plagued with the issue of the durability of the anti-tumor response of autologous TCR-Ts, and a way to overcome this obstacle was lymphodepletion before infusion of TCR therapy [22,52,53]. A study by Kageyama et al., 2015, focused on targeted HLA-A*24:02-restricted MAGE-A4-expressing esophageal tumor cells in 10 patients via TCR-Ts without any lymphodepletion and showed an increased survival length of transferred TCR-Ts but with limited to no tumor regression [49]. One benefit of lymphodepletion before the infusion of TCR-T cells is that it increases cytokine release due to the depletion of lymphoid cells. This enhanced cytokine release stimulates a stronger antigen-presenting cell (APC) response and improves the availability after the previous T cells have been removed [49,54]. Another strength of lymphodepletion is that it helps eliminate regulatory T cells, which can suppress immune response to cancer cells, allowing for an environment that helps improve the efficacy of TCR therapy [55]. Besides utilizing the benefits of lymphodepletion, modulating the mechanisms that assist with T cell peptide recognition helps ensure T cell survival, and this is important, as continuous self-pMHC (peptide major histocompatibility complex) interaction is what maintains the longevity of the TCR response [56]. This concept is explored in a study by Zhao et al., 2007, where it was concluded that T cell recognition is influenced by the number of p/MHC complexes available for binding, the affinity of the TCR for the MHC via co-receptors (CD8) and the affinity of the TCR for the target peptide [4,57]. Taking these factors into account, the first challenge in targeting an antigen with TCRs is that these antigens have to be presented on specific HLA molecules and the process of finding targets of interest involves immune-informatics techniques (artificial neural networks and the determination of quantitative structure–activity relationships), which analyze each step of the intracellular processing of the antigen. Processing pathways of MHC I antigens involve pMHC binding, cleavage of the antigen intracellularly and the specific transporters moving the antigen from the cleavage site to the cell membrane [58,59]. The utilization of these immuno-informatics methods, particularly involving neural networks, did not prove to be efficacious when identifying the MAGE A4-derived peptide GVYDGREHTV [48]. Understanding the processing pathway of MHC 1 antigens is important when considering a target for TCR binding because processing can differ between mouse models and humans [58,59]. Attention to the binding dynamics involved in TCR therapy and understanding the threat of cross-reactivity are principles that were applied in the development of the TCR therapy targeting MAGE A4.

## 3. Current MAGE A4 T Cell Therapy Trials

Currently, the most successful attempts to target the MAGE A-4 CTA for therapeutic effects have been performed via genetically engineered TCR therapy [60,61,62,63]. A summary of the trials involving targeted MAGE-A4 therapy is presented in Table 1. The trials included in Table 1 focus not only on TCR-T therapies specific to MAGE A4 but also mention some bispecific antibody approaches (NCT05129280 and NCT03973333) and combination-therapy options with immune checkpoint inhibitors (NCT04408898) [64,65]. The most promising trials belong to the engineered TCR-specific MAGE A4 group; such trials include NCT04044768, NCT03132922 and NCT02096614, which are active studies with ongoing recruitment, and some with in vivo human data, where it is available, compared to bispecific approaches targeting MAGE A4 that do not have a large robust clinical population [21,61,66,67,68]. Furthermore, Table 1 also mentions a combination-therapy trial (NCT04408898) which combines ADP-A2M4 and pembrolizumab with the aim of enhancing treatment efficacy by targeting multiple pathways involved in tumor growth and immune evasion. This modality did not demonstrate early promise in the development stage compared to engineered TCR autologous therapy [69]. Overall, the table represents the current landscape of targeted MAGR A4 cellular therapies that are in development.

As mentioned earlier, some of the greatest advances have been observed in autologous TCR therapy targeting MAGE A4+ tumor types in HLA-A*02-restricted patients [49,61,63,70]. A notable TCR therapy developed by Adaptimmune called ADP-A2M4 (also known as Afamitresgene autoleucel (afami-cel)) is an HLA-A2-restricted treatment targeting the MAGE-A4 peptide GVYDGREHTV [48]. This engineered TCR is transduced into autologous T cells via a lentiviral vector and then expanded in vitro before being reinserted back into the patient [71]. The early stages of the ADP-A2M4 development involved screening for any possible cross-reactivities by incorporating stringent testing with various induced pluripotent stem cells (iPSCs), and ADP-A2M4 showed a very low-level reaction with the MAGE A4-airway epithelial cell line, which on repeat testing was not replicated [48]. The in vitro testing of ADP-A2M4 was performed not only on mouse xenograft melanoma cell lines but also on 3D microtissues, which are cell lines that have the ability to polarize and differentiate around a protein scaffold, enabling replication with greater similarity to the in vivo tumor microenvironment [48,72]. The ADP A2M4 TCR had alloreactivity toward HLA-A*02:05 and showed a strong response to several different cell types, meaning this response could not be elicited from one specific peptide. Further workup was also performed to search for ADP A2M4 cross-reactivity with other MAGE family proteins through a protein-sequence alignment program, which showed the TCR to have very weak affinity toward MAGE A8- and B2-expressing malignancies. There was no concern about severe cross-reactivity, as MAGE B2 expression is isolated to the placenta and testis and MAGE B8 is restricted to the brain. Binding to these CTAs is very weak in ADP A2M4 exposure to B cell precursor leukemia cell lines (NALM6) transduced with these peptides, and qrt PCR (real-time PCR) of these homologous CTA peptides was run against a tissue biobank showing no issues with off-target tumor cell expression [48]. Thus, overall, cross-reactivity with ADP A2M4 seemed to be depressed variably.

In the early development of the ADP A2M4 TCR, it showed considerable anti-tumor activity when tested in in vitro models and was eventually utilized in a phase 1 clinical trial (NCT03132922), where it demonstrated an ORR of 44% for SS and seven partial responses in patients with SS [48,61,62]. In fact, two patients with extensive SS (as indicated by the progression of pulmonary and pleural metastases on computed tomography) both showed partial responses (PRs) to the ADP-A2M4 treatment, with a time to tumor response (TTR) of 6 weeks. Interestingly, when a second infusion of ADP-A2M4 therapy was given, it did not demonstrate a response for one of these SS patients, and instead the tumor showed a lower MAGE A4 expression compared to their first infusion. Ultimately, this phase 1 clinical trial (NCT03132922) showed no serious T cell-mediated toxicity but did show grade 3 or higher adverse effects like anemia, neutropenia and thrombocytopenia [61]. Unfortunately, there were two patient deaths reported, secondary to aplastic anemia and cerebral vascular accidents. The success in this phase 1 trial (NCT03132922) led to a phase 2 trial (SPEARHEAD-1, NCT04044768), with the ADP-A2M4 infused into 44 patients with SS and 8 patients with MRCL expressing MAGE-A4 and having the HLA-A2 haplotype [73]. SPEARHEAD-1 (NCT04044768) demonstrated an ORR of 37% (in 19 of 52 patients), with a median duration of response of 11.6 months and a median progression-free survival of 3.7 months [73]. These higher response rates were only seen in female patients who had higher MAGE-A4 expression compared to patients who did not utilize bridging therapy and those who came into the trial with initially smaller malignant tumor sizes [63,73]. All 52 of the patients were shown to have cytopenia as a common treatment adverse event, but after week 12 of the ADP A2M4 infusions the cytopenia resolved [73]. Besides cytopenia, 51 out of 52 patients experienced grade 1 or 2 cytokine release syndrome (CRS) [73]. In terms of deaths related to treatment, there were 0 in this trial, and all 28 deaths reported in this trial were related to disease progression [73]. Based on these data, on August 02, 2024, the FDA granted accelerated approval of afamicel for adults with unresectable or metastatic SS who have received prior chemotherapy; are HLA-A*02:01P, -A*02:02P, -A*02:03P or -A*02:06P-positive; and whose tumor expresses the MAGE-A4 antigen as determined by FDA-approved or -cleared companion diagnostic devices [74].

Though the ADP A2M4 TCR demonstrated excellent effectiveness against certain MAGE A4-associated tumors in recent trials, further advances in the development of the ADP A2M4 TCR treatment which involved adding CD8 co-receptor activity helped broaden the overall functionality of this immunotherapy [75,76]. In particular, the SURPASS-2 trial (NCT04752358) involved 13 patients with esophageal cancer, gastric cancer and esophagogastric junction adenocarcinoma exposed to ADP-A2M4CD8 in September 2022, which resulted in two PRs and eight SDs [77]. This updated version of the compound is now under clinical investigation (NCT04752358 and NCT04044768) for ovarian and esophagogastric cancers [68,78].

## 4. Development and Application of Affinity-Enhanced Autologous MAGE A4 TCR Therapy via the CD8 α Co-Receptor

The cornerstone of T cell activation involves CD8 and CD4 glycoproteins that enhance the ability of T cells to recognize MHC class 1 and 2 molecules on APCs or target cells. While it is important to understand the interaction between T cells and pMHC in TCR therapy, the relationship between the CD8 and CD4 co-receptors with pMHC is something that can be utilized to enhance TCR therapy [79]. Most adaptive cell therapies rely on CD8 T cells for their cytotoxic functionality; however, there is a growing body of evidence suggesting that utilization of CD4 cells is also needed because it enables more widespread TAA targeting and increases the longevity of the CD8 T cell response [80,81]. CD4 cells are also responsible for directing the secretion of cytokines that enable tumor cell infiltration and assist in the creation of memory CD8 T cells [82,83]. Most solid tumors usually express pMHC-1, which means that natural CD4 T cells usually cannot recognize them without the addition of a pMHC-1 TCR and a CD8 co-receptor [84].

**Figure 1:**
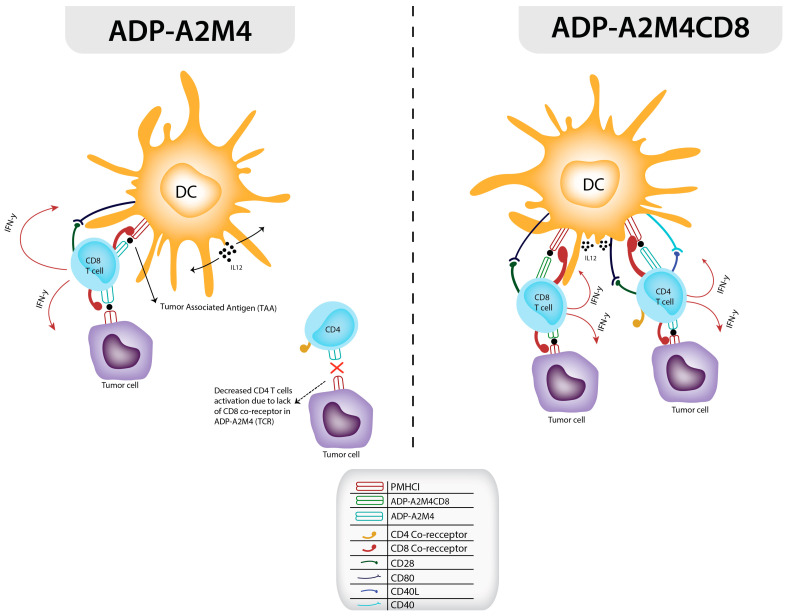
In this figure, the limited T cell activation is demonstrated with the ADP A2M4 TCR with the pMHC-1. Normally, CD4 T cells have limited activation with pMHC-1 because of the lack of CD8 co-receptor. With the ADP AM2M4CD8 TCR, not only are CD4 T cell now secreting granzymes and IFN-γ but it also upregulates other CD8 T cell activation via increasing dendritic cell activity which release IL-12 and CD80 expression indirectly enabling greater CD8 T cell activity [76].

The significance of the CD8 co-receptor is highlighted by a study conducted by Purbhoo et al. (2001). In this study, a point mutation was introduced into HLA A2 presented by antigen-presenting cells (APCs), disrupting the CD8 co-receptor’s ability to bind to the TCR/pMHC complex. This disruption led to reduced T cell activation, regardless of how much the antigen concentration was increased [85]. There have been great strides in improving CD4+ cytotoxic activity via affinity genetic enhancement of the TCR with the addition of the CD8 alpha co-receptor [75]. In one method, the idea of utilizing the CD8 co-receptor in TCR-Ts is combined with the immune roles of CD4 and CD8 T cells in a synergistic anti-tumor response. The cytotoxic effects of CD8 T cells depend on CD4 T cells, which activate them with interleukin-2, and, recently, with IL-21 as well [86,87]. Naturally, CD4+ T cells have very low cytotoxic abilities; however, a TCR can be transduced into them, which can enable them to interact with MHC 1 and 2 receptors, but even with this, they cannot yield a fully effective T cell response without the CD8 co-receptor interaction (illustrated in Figure 1) [88]. The efficacy of the addition of the CD8 co-receptor to an existing CD4+ TCR was evaluated in a study by Anderson et al., which compared their CD4+ ADP-A2M4 targeting MAGE A4 in HLA-A*02-restricted patients with CD8 (ADP-A2M4CD8) [76]. This study was based on a previous approach of Willemsen et al., which had created TCRs with CD8 alpha co-receptors with affinity for MAGE-A1 melanoma cells in HLA A1-restricted patients. The Willemsen et al. study substantiated the fact that primary t-TCRs with CD8 α and primary CD4 t-TCRs with Cd8 α are both necessary for prolonging the cytotoxic effectiveness of CD8 TCR-transduced cells and encourage in vivo production of CD8 effector cells targeting tumors [89,90]. A similar response was seen in the Anderson et al. 2023 study, which increased IFN-γ and IL-12 detection in ELISA assays from MAGE A4 HLA-A2-restricted melanoma cells lines via ADP-A2M4CD8 TCRs compared to TCRs without the CD8 α receptor [76]. The success seen in the in vivo studies carried out by Anderson et al. led to the eventual utilization of ADP-A2M4CD8 TCRs targeting MAGE-A4 in clinical trials [67,76].

**Figure 2:**
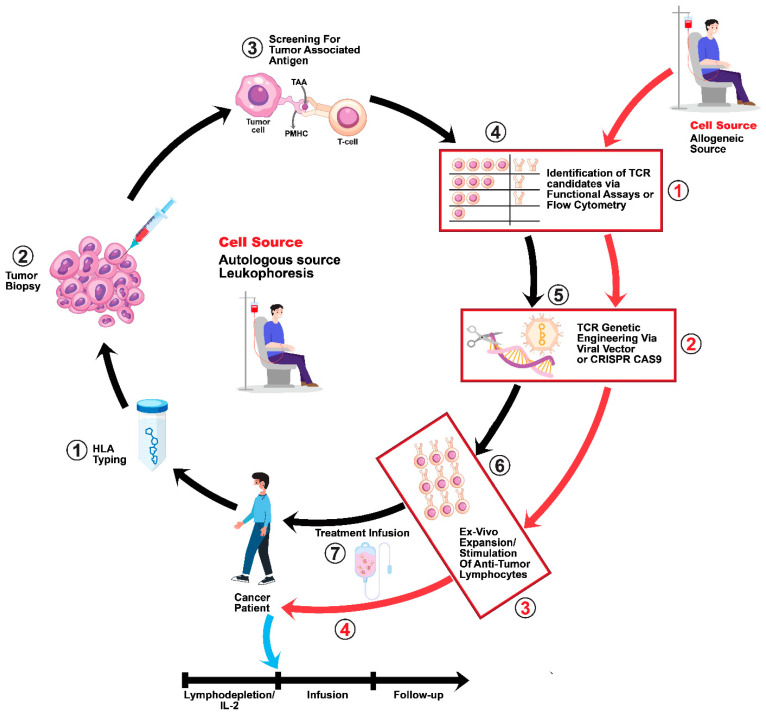
Cancer patients are first screened for HLA type, and once this is confirmed a tumor biopsy is performed to screen for correct TAA presentation. Once the TAA presence is established, potential TCR candidates can be developed by isolating TILs from patients that are capable of binding to the TAA. These TILs will have their TCR genetic sequences examined via next-generation sequencing, enabling the creation of high-affinity-binding TCRs. Once these high-affinity-binding TCRs have been isolated, they can be delivered to T cells via viral methods like a lentivirus-based vector or non-viral methods like CRISPR-Cas9 technology. If the autologous route is preferred, then PBMCs (peripheral blood macrophages) are obtained from the cancer patient via leukapheresis. If an allogenic approach is taken, then PBMCs are obtained from a healthy patient. Once a T cell source is obtained, these cells can be stimulated with anti-Cd3 and anti-Cd28 antibodies and then transduced with the high-affinity engineered TCRs [94,95].

One of the earliest clinical trials of ADP-A2M4CD8 TCR was the SURPASS trial, and it started in 2019 with five patients who had various tumor types restricted to the HLA-A*02 haplotype and who were exposed to ADP-A2M4CD8 along with nivolumab or pembrolizumab [67,76]. Within the first year of the SURPASS trial, two patients showed a stable response and the other three showed OR and SD [67]. Fast-forward to March of 2023, by which time the SURPASS trial had a total of 51 patients enrolled and an OR of 35.6%, with 14 PRs and 2 CRs [91]. The ongoing SURPASS-3 trial (NCT05601752) is exploring the use of ADP-A2M4CD8 in combination with Nivolumab for HLA A2-restricted patients with MAGE A4-positive ovarian cancer [92]. Ultimately, the addition of the CD8 co-receptor to this autologous T cell therapy by Adaptimmune has proved to be advantageous; however, there are underlying disadvantages compared to allogeneic bispecific T cell-engaging (BiTE) therapies [67,77,91]. The autologous TCR approach (as can be seen in Figure 2) has quite a few delays in processing compared to allogeneic BiTE therapy; for instance, a specific number of leukocytes is required for a successful leukapheresis; the T cells have to be transduced via lentiviral vectors; and, finally, there is a wait for successful T cell expansion in vitro [93]. The BiTE therapy route allows for more frequent and precise dosing of therapy compared to the TCR approach, and the antibodies are easily manufactured in large batches, contrasting favorably with the singular dosing and 6–8 week total processing time of adoptive T cell therapy [93].

## 5. Allogeneic Bispecific MAGE A4 BiTE Therapy

Allogeneic bispecific cell therapy is an off-the-shelf solution that offers not only a more practical approach to manufacturing and time to administration, but also, by both targeting the TAA and recruiting immune cells to the tumor site, it allows for a more robust therapeutic response [92,93]. Bispecific T cell therapy essentially engages with TAA and with CD3 on the TCR complex. An advantage of this therapy is that it encourages the formation of lytic synapses between cytotoxic T cells and their target cells, allowing an effective immune response [96]. Another feature of this therapy is how the TAA binding domain of the BiTE can be engineered to incorporate a greater number of binding domains, allowing for greater anti-tumor activity [97,98]. These molecular advantages and efficient manufacturing benefits were demonstrated in the first approved BiTE therapy, Blinatumomab, which was used to treat adult patients with B-ALL (precursor B cell acute lymphoblastic leukemia). In the randomized phase 3 TOWER study, Blinatumomab was shown to have a higher complete response rate and minimal residual disease negativity compared to traditional chemotherapy used in Ph-negative precursor B-ALL patients [99]. The success seen with the BiTE Blinatumomab was also seen with different BiTEs in other hematological malignancies like multiple myeloma, acute myeloid leukemia (AML) and B cell non-Hodgkin lymphoma (RO7082859, NCT03145181 and NCT02152956) [98,100,101]. BiTE therapy has been shown to have greater success in hematological malignancies compared to solid tumors, which possibly could be explained by the lack of antigen presentation on the surfaces of solid tumors. Attempts to overcome the scarcity of TAA presentation on tumor surfaces by increasing therapeutic doses has not been successful, as seen with the BiTE catumaxomab. In the phase 1 clinical trial of catumaxomab, as doses reached therapeutic levels, there was an increase in side effects like liver toxicity and diarrhea [102]. Another important consideration is that, to date, most BiTEs have targeted extracellular antigens. However, there have been advancements in developing BiTEs aimed at intracellular TAAs presented by HLA molecules. Examples include gp100, PRAME and NY-ESO-1, which are currently being tested in clinical trials (NCT04262466, NCT03070392 and NCT03515551) [103,104,105,106,107]. In the pursuit to target intracellular antigens more efficiently, a type of BiTE therapy called ImmTAC (immune-mobilizing monoclonal TCRs against cancer) has been pursued.

ImmTAC therapy consists of a soluble monoclonal TCR (mTCR) bound to a CD3 single variable chain fragment [108,109]. The ImmTAC approach showed excellent specificity and sensitivity in in vivo xenograft models which targeted several different TAAs like MAGE A4, NY-ESO-1 and gp100. In fact, this efficacy can be seen in Tebentafusp, an ImmTAC that targeted the gp100 TAA in HLA-A2*01-positive patients with metastatic uveal melanoma; in a phase 3 clinical trial, it showed an increase in OS (9%) compared to the control group (5%), which received guideline-directed first-line therapy [110]. The efficient sensitivity of ImmTAC therapy stems from the utilization of affinity-matured mTCRs. The idea of using affinity-matured mTCRs is that the targeted pMHC molecule tends to naturally present in lower concentrations in solid tumors, and, ultimately, the study by Liddy et al. deduced that the overall potency of this therapy hinges on how strong the interaction is between the mTCR and the pMHC [108]. There are also a few ImmTACs targeting MAGE A4 tumors which are in development or active in clinical trials (NCT03973333) [111,112,113,114]. There are two allogeneic bispecific trials referenced in Table 1, with one still active involving IMC-C103C (NCT03973333) [112,113]. IMC-C103C is an ImmTAC targeting HLA-A*02-positive patients with ovarian carcinoma (OC) (87% platinum resistance) with variable MAGE A4 expression, which was tested in a phase 1 dose escalation trial. There were 15 patients who were MAGE A4-positive, and in that group 5 had SD and 1 had PR. A limiting factor in this trial was probably that a greater number of patients with OC were pretreated and had very low MAGE A4 expression [115]. Along with IMC-C103C, there is another BiTE called CDR202 currently in development, which also targets MAGE-A4 tumor cells in mice which are HLA-A*02:01-positive. It was shown to have excellent target cell lysis as well as no cross-reactivity when tested against HLA A*02 primary cells. There are tentative plans to use this BiTE for a multi-tumor phase 1 trial in 2024, targeting patients with the HLA-A*02 haplotype and MAGE-A4-positive tumors [111]. Though ImmTACs seem promising, being under the BiTE umbrella exposes them to similar challenges faced by allogeneic bispecific therapy in general, which include increased prevalence of serious adverse events and low response rates in most solid tumors. For example, in the phase 3 trial for Blinatumomab, 62% of patients experienced serious adverse events like CRS (cytokine release syndrome), neurotoxicity and neutropenia; however, dose adjustments through the trial assisted in reducing such events [99]. Another challenge contributing to the low response rates of bispecific therapies in solid tumors is the immunosuppressive nature of the tumor microenvironment. This environment often includes myeloid-derived suppressor cells, suppressive cytokines, T regulatory cells and other factors that inhibit immune responses. These elements not only hinder the effectiveness of bispecific therapies but also pose difficulties for TCR and CAR-T therapies [116]. Methods have been developed to overcome this immunosuppressive environment via BATs (bispecific antibodies with armed T cells), which take the patient’s T cells and stimulate them ex vivo with anti-CD20 and anti-CD3 and inject them back into the patient with the bispecific therapy [117,118]. If the issues regarding dosage and solid tumor response can be overcome, allogeneic bispecific therapy could prove to be an effective option in the future compared to autologous adoptive T cell therapy.

## 6. Current Challenges and the Future Outlook for Autologous TCR Immunotherapy

The hurdles faced by autologous TCR therapy are centered around off-target binding, HLA-restricted binding, the in vitro production of T cell products, mispairing of exogenous TCR chains introduced via lentiviral transduction and toxicities involved in the induction of such therapy. An example of the dangers of off-target binding was seen with the MAGE A3 TCR, which had off-target binding to titin, a myocyte protein, causing the death of two patients in a clinical trial [119]. The off-target binding was not discovered initially because normal undifferentiated cardiac HLA A01-positive cells were used and showed no cross-reactivity with the MAGE A3 TCR in an IFN-gamma assay. However, when more biologically relevant cardiac cells were used (an I-cell culture system that consisted of a mixture of spontaneously electrically active atrial, nodal and ventricular-like myocytes), it showed very potent activity in the IFN-gamma assay [119]. Another study with a MAGE A3 TCR showed off-target binding to the brain, as the TCR that was used had epitope recognition for MAGE A12, which is present in the human brain. Three out of nine patients who died as a result of this off-target binding had developed Parkinson-like symptoms, and autopsies demonstrated leukoencephalopathy [120]. Again, there are many dangers associated with off-target binding, which has led to more TCR therapy studies being focused on the development of stringent in vitro testing techniques that are closer to physiological conditions. For example, the study by Davari et al. had sought to utilize in vitro models (consisting of LCLs (lymphoblastoid cells) and tumor lines that were HLA A2-positive and MAGE A4-negative) particularly to observe off-target binding of their MAGE A4 TCRs to various HLA allotypes [75]. There have also been in silico advances in harnessing HLA cross-reactivity in TCR therapy, using bioinformatic methods such as NetTCR and TCRGP (TCR Gaussian Process) [121,122].

One barrier faced by TCR therapy is due to its limited applicability to only certain populations with specific HLA types. Most studies have shown HLA A 02 restriction to be more prevalent in Caucasians compared to Asians and African Americans [123]. Currently, there is a certain genetic engineering technique that targets synovial sarcoma and does not require HLA matching which utilizes aAVCs (artificial adjuvant vector cells) in order to accomplish this task. The aAVCs are essentially cells filled with targeted TAAs. What makes aAVCs unique is that their surfaces are filled with CD1d-presenting molecules which contain a galactosylceramide (a-GalCer) ligand. The aGalCer ligands are what stimulate natural killer cells, which then lyse the aAVC cells and release the TAAs, which are taken up by the patient’s APCs. The APCs then stimulate CD4/CD8 cells, activating the cytotoxic pathway. Currently, a phase 2 trial for this technology is being conducted on patients with NSCLC and has shown prolonged overall survival (UMIN000007321) [124,125].

Besides limitations to TCR therapy due to HLA restriction, there are certain obstacles facing the production of in vitro T cell products as well as the side effect profile of the lymphodepletion regime before administering said product [99]. Before the application of in vitro T cell products, patients must undergo lymphodepletion to receive TCR therapy. It is also known that intensification of lymphodepletion improves response to TCR therapy but at the cost of increased toxicity [126,127]. This was especially true in a trial by Adaptimmune, where a patient cohort with lower NY-ESO-1 intra-tumoral antigen expression showed poor clinical responses if they did not have an intense lymphodepletion regimen consisting of fludarabine with cyclophosphamide (NCT01343043) [128,129,130]. Lymphodepletion has been shown in some studies involving CAR-T therapy to cause CRS; however, the lack of uniform reporting for after-effect criteria makes this association controversial [131]. The type of lymphodepletion regimen used in certain CAR-T studies also affected the rate of CRS, as a greater frequency of CRS was observed with lymphodepletion regimens consisting of cyclophosphamide or fludarabine [130]. On top of side effects seen during the induction period before administering TCR therapy, there were also challenges in the creation of the TCR via lentiviral transduction.

Lentiviral vectors are usually utilized in TCR gene transfer, as they are able to transduce into non-dividing cells with a high degree of stability [132]. Normally, T cells express a single TCR composed of a specific alpha and beta chain, which dictates their antigen specificity. When exogenous TCR chains are introduced via lentiviral transduction, these new chains have the ability to pair not only with each other but also with the existing endogenous TCR chains, leading to unpredictable antigen specificities [133]. There are several mechanisms that have been designed to help combat this mispairing event that involve precise gene-editing techniques, such as CRISPR-Cas9 and TCR chain modifications. CRISPR-Cas9 can be utilized to knock out the endogenous TCR genes or insert the exogenous TCR genes into specific loci, minimizing the chances of mispairing [134]. Another method utilized to avoid this error is modifying the TCR chain itself by either replacing human constant regions of the TCR alpha and beta chains with murine constant regions, preventing the mispairing seen with endogenous human TCR chains, or further stabilizing the biochemical structure of the TCR heterodimer by adding another disulfide bond between the constant regions of the TCR alpha and beta chains [135]. On top of the issues faced with lentiviral vector gene transduction, there is also the tenuous nature of the culturing conditions during the T cell differentiation process that play an important role in TCR therapy production.

Culturing conditions during T cell expansion ex vivo play a critical role in shaping the composition of the final product, impacting both the memory phenotype and the CD4/CD8 ratio [136]. Studies in animal models have shown that having a higher percentage of memory and naive T cells compared to T effector cells leads to a more durable therapeutic response compared to those with higher percentages of T effector cells [137,138]. One clinical trial (NCT00586391 and NCT00709033) demonstrated that CAR-T therapy in B cell lymphoma patients showed greater in vivo expansion when the infused product was primarily composed of memory T cells compared to products with a lower proportion of memory T cells [139]. An optimal CD4/CD8 ratio is thought to promote a more balanced and effective immune response. CD4+ helper T cells provide crucial support to CD8+ cytotoxic T cells, enhancing their ability to eliminate target cells. Conversely, an imbalance in the CD4/CD8 ratio can have detrimental effects. For instance, a skewed ratio towards CD8+ T cells might increase the risk of toxicity, while a predominance of CD4+ T cells might compromise the cytotoxic potential of the therapy. Therefore, optimizing the ex vivo culture conditions to achieve a desired CD4/CD8 ratio is essential for maximizing the therapeutic benefit and minimizing potential adverse effects. Ultimately, obtaining a greater memory T cell subset comes down to the time spent in the proliferative phase in production along with the choice of cytokine exposure. Most production involves exposure to IL-2; however, there are studies showing that combinations of cytokine exposures, like IL-7 and IL-15, help to create a more robust T cell memory composition in the infused product [139,140,141]. Though culturing conditions prove to be an obstacle in the creation of TCR therapy, this immunotherapy faces significant challenges regarding scalability and affordability.

When it comes to this type of autologous immunotherapy, each treatment requires a tailored process involving the isolation and modification of a patient’s own T cells. This individualized approach is inherently complex, time-consuming and resource-intensive, limiting the number of patients who can be treated, and this has been described above with regard to the HLA haplotype restrictions [142]. The manufacturing process involves multiple intricate steps, including T cell isolation, genetic modification, expansion and quality control (as depicted in Figure 2). These steps require specialized equipment, skilled personnel and stringent quality control measures, adding to the overall cost. The materials and reagents used in the manufacturing process, including viral vectors, cytokines and culture media, are expensive, further contributing to the high cost of the therapy. As discussed earlier, the option of allogeneic or “off-the-shelf” TCR-T cell products that can be used for multiple patients without the need for individual manufacturing could significantly improve scalability and reduce costs [143].

Though there are a multitude of issues plaguing TCR therapy, there are solutions currently being explored to improve its overall therapeutic response. The future direction of targeting multiple antigens is something to strongly consider in TCR therapy. A study by the Baylor College of Medicine (NCTNCT01333046) demonstrated therapeutic responses by utilizing this approach when using TCR therapy targeting five tumor-associated antigens in patients with lymphoma. In this study, there were 15 patients with chemorefractory lymphoma, with 6 out of the 15 entering durable complete remission [144]. In addition, influencing the local tumor microenvironment has been another way of boosting the functionality of TCR therapy, one aspect of which is the influence of immune checkpoint proteins such as PD-1 (program death ligand 1), which have been shown to prevent T cell activation. In fact, a study utilizing NY-ESO-1 TCR therapy was able to augment their functionality by inhibiting the PD-1 checkpoint [145,146]. Immune cells themselves also have the ability to influence the tumor microenvironment by the cytokines they secrete, such as IL-2, which has been shown to improve the survival of T cells [147]. Though there are various points of improvement in TCR therapy as a whole, MAGE A4 TCR therapy itself has been shown to have a lower cross-reactivity profile compared to other TCR CTAs available [67,68,75,76,119].

## 7. Conclusions

Autologous TCR therapy, particularly for MAGE A4, shows much promise for the future. Results from clinical trials (NCT03132922, NCT04044768 and NCT04044859) [73,77,91] continue to show robust outcomes, particularly in patients with synovial sarcoma, as reflected in the recent FDA approval [67,68,76,92,148]. The genetic engineering methods used to develop and improve the targeting of ADP-A2M4 and ADP-A2M4CD8 have continued to improve T cell responses and durability. Larger barriers to overcome include developing more robust in vitro testing regimens, which is something already being undertaken, as seen in the development of ADP-A2M4CD8 [75,76]. As additional MAGE A4 studies provide results, more clinical data will become available to enhance strategies for minimizing cross-reactivity, improve efficacy within the tumor microenvironment and optimize TCR and pMHC interactions. Moreover, allogeneic alternatives are also being tested in the clinic, with bispecific T cell engagers, for instance, offering a promising off-the-shelf option for patients in the near future.

## Figures and Tables

**Table 1:** Here is a table describing ongoing and past immunotherapies targeting MAGE A4.

**NCT Number**	**Sponsor**	**Status and Study Dates**	**Phase**	**Tumor Type**	**Intervention**	**Therapy Type**	**Outcomes**
NCT04044768	Adaptimmune	Start: 8/13/19 Stop: 10/10/2021	Phase 2	Synovial sarcoma and myxoid/round cell liposarcoma	Genetic: afamitresgene autoleucel (previously, ADP-A2M4)	Autologous	ORR: 34% (95% CI, 20.86–49.31%), SD: 51.1% ORR in SS: 35.9% ORR in MRCLS: 25.0% PFS: 8.7 months
NCT03132922	Adaptimmune	Start: 5/15/17 Stop: 12/27/2022	Phase 1	Urinary bladder cancer, melanoma, head and neck cancer, ovarian cancer, non-small cell lung cancer, esophageal cancer, gastric cancer, synovial sarcoma, myxoid round cell liposarcoma, and gastroesophageal junction cancer	A4c1032-T cells combined with low-dose radiation	Autologous	(No CR, all PRs in SS) PR: 25% SD: 39% PD: 17.8% NE: 17.8%
NCT05129280	Hoffmann-La Roche	Terminated due to program discontinuation	Phase 1	Solid tumors	RO7444973	Allogeneic, bispecific T cell engager of MAGE-A4 and CD3	Not available
NCT03973333	Immunocore Ltd.	Ongoing Start: 5/17/19	Phases 1 and 2	Ovarian carcinoma	IMC-C103C in combination with Atezolizumab	Allogeneic, bispecific T cell engager of MAGE-A4 and CD3	15 pts MAGE A4 + PR: 1/15 SD: 5/15 Pretreated OC w/low MAGE-A4
NCT05601752	Adaptimmune	Ongoing Start: 06/26/23	Phase 2	Ovarian cancer	ADP-A2M4CD8 cells with Nivolumab	Autologous	Not available
NCT04752358	Adaptimmune	Ongoing Start: 2/12/23	Phase 2	Esophageal cancer and esophagogastric junction cancer	ADP-A2M4CD8	Autologous	Not available
NCT05642455	Adaptimmune	Ongoing Start: 6/30/23	Phases 1 and 2	Synovial sarcoma, malignant peripheral nerve sheath tumor (MPNST), neuroblastoma and osteosarcoma	Afamitresgene autoleucel	Autologous	Not available
NCT02096614	Mie University	Start: 04/2014 Stop: 03/2021	Phase 1	Solid tumors	TBI-1201 Drug: Cyclophosphamide Drug: Fludarabine	Autologous	Not available
NCT01694472	Tianjin Medical University Cancer Institute and Hospital	Start: 07/2012 Stop: 12/2016	Phase 1	Solid tumors	MAGE-A4 TCR gene-modified T cells	Autologous	Not available
NCT04408898	Adaptimmune	Withdrawn Start: 07/02/2020 Stop: 12/2021	Phase 2	Head and neck cancer	ADP-A2M4 in combination with pembrolizumab	Autologous	Not available
NCT04199559	Henan Cancer Hospital	Start: 12/01/2019 Stop: 01/01/2022	Phases 1 and 2	Non-small cell lung cancer	Autologous dendritic cells pulsed with antigen peptides and nivolumab	Autologous	Not available
NCT01333046	Baylor College of Medicine	Ongoing Start: 01/2012	Phase 1	Hodgkin and non-Hodgkin lymphoma	MultiTAA T cells and azacytidine	Autologous Expanded polyclonal T cells reactive to five TAAs: PRAME, SSX2, MAGEA4, SURVIVIN and NY-ESO-1	Not available
NCT03247309	Immatics US, Inc.	Ongoing Start: 12/19/2018	Phase 1	Head and neck cancers and lung cancer	IMA201	Autologous	Not available

TAA: tumor-associated antigen; PFS: progression-free survival; ORR: objective response rate; OS: overall survival; DoR: duration of response; PD: progressive disease; SD: stable disease; NE: not evaluable.
